# Supplementary material for: Transsaccadic integration operates independently in different feature dimensions
Source: J Vis. 2021 Jul 15;21(7):7. doi: 10.1167/jov.21.7.7 (PMC8288057; doi:10.1167/jov.21.7.7)
Supplement: Supplement 1 [file jovi-21-7-7_s001.pdf]

Supplementary Materials

Model fits for the object-based integration model

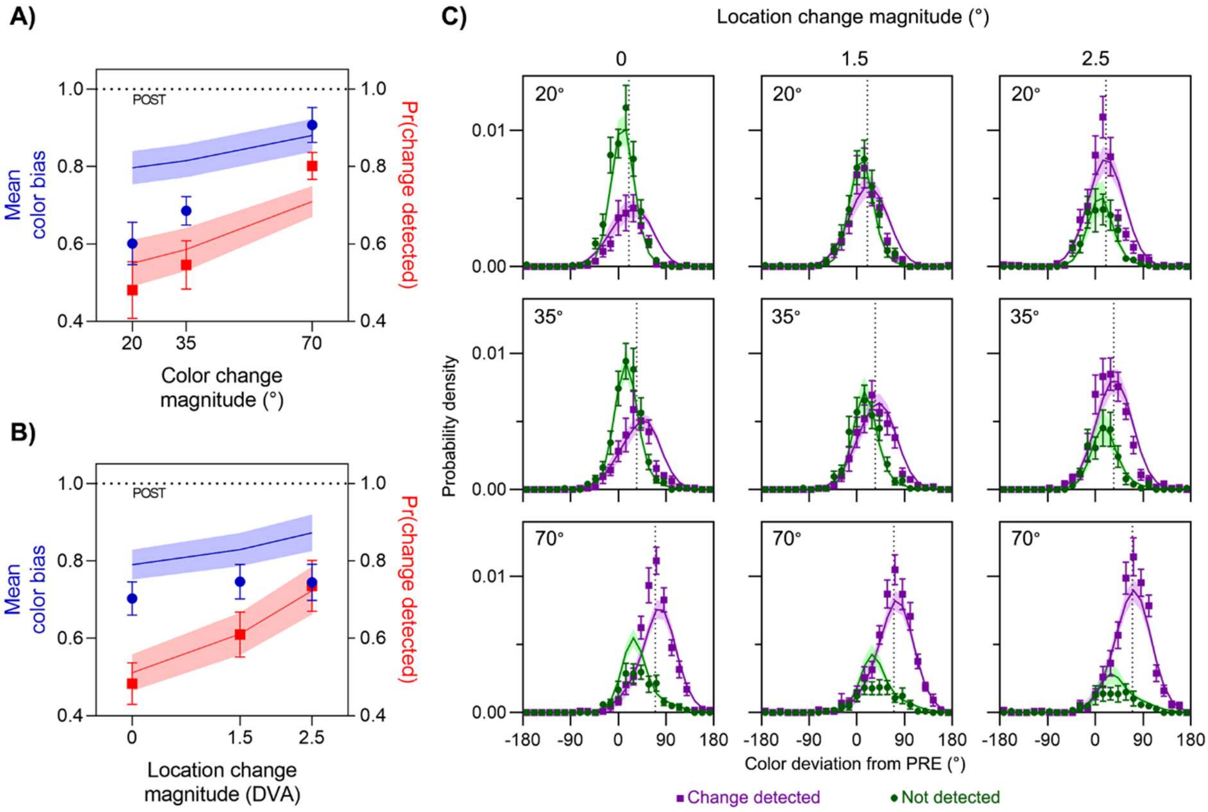

Figure S3. Results of Experiment 1. (A & B) Mean color bias (blue symbols, left y-axis) and mean frequency of detecting a change (red symbols, right y-axis) are plotted as a function of the magnitude of (A) color change and (B) location change. (C) Distribution of reported color relative to pre-saccadic color (0°) and post-saccadic color (dotted vertical line in each panel), plotted separately for trials where a change was detected (purple) and not detected (green). Areas under each distribution reflect the frequency of detection. Each panel corresponds to a different pairing of color change (rows: magnitude indicated in top-left of each panel) and location change (columns: magnitude indicated at top). Error bars and error patches represent ± 1 S.E.

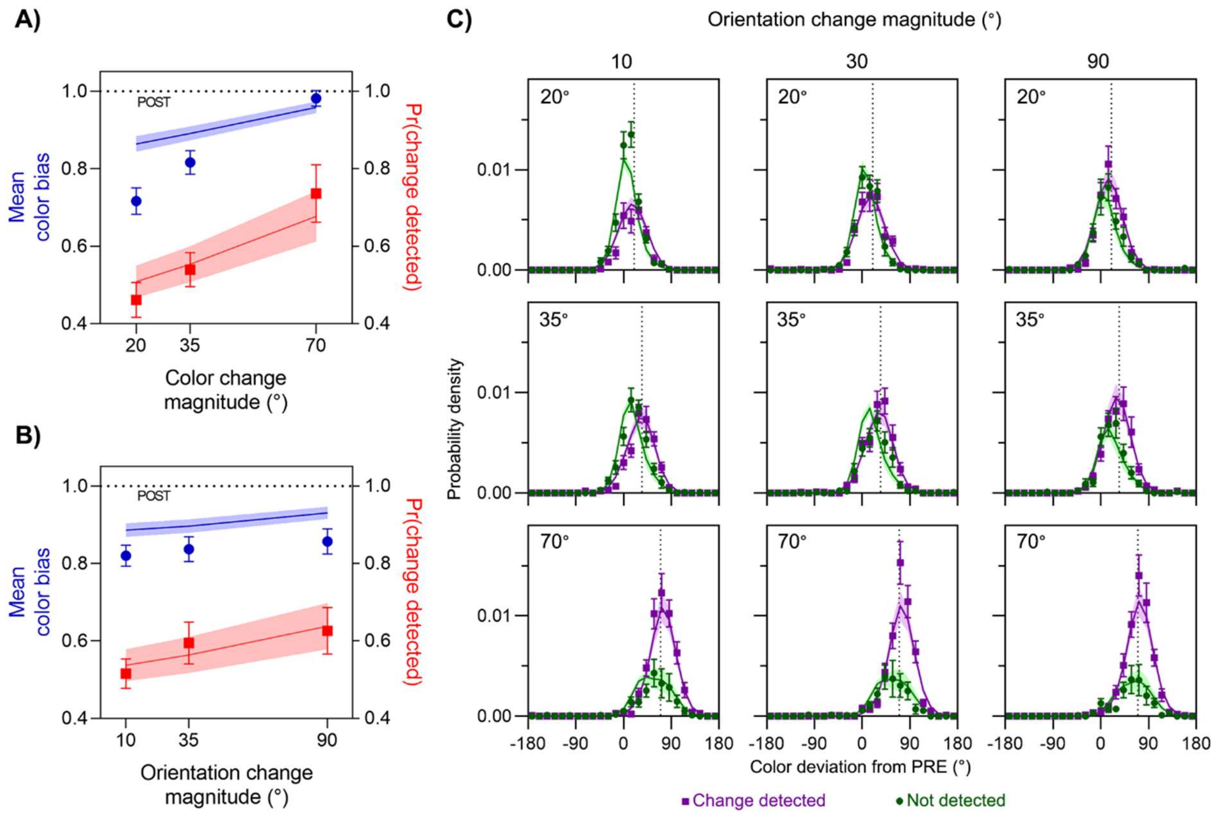

Figure S4. Results of Experiment 2. (A & B) Mean color bias (blue symbols, left y-axis) and mean frequency of detecting a change (red symbols, right y-axis) are plotted as a function of the magnitude of (A) color change and (B) orientation change. C) Distribution of reported color relative to pre-saccadic (0°) and post-saccadic color (dotted line in each panel), plotted separately for trials where a change was detected (purple) and not detected (green). Areas under each distribution reflect the frequency of detection. Each panel corresponds to a different pairing of color change (rows: magnitude indicated in top-left of each panel) and orientation change (columns: magnitude indicated at top).

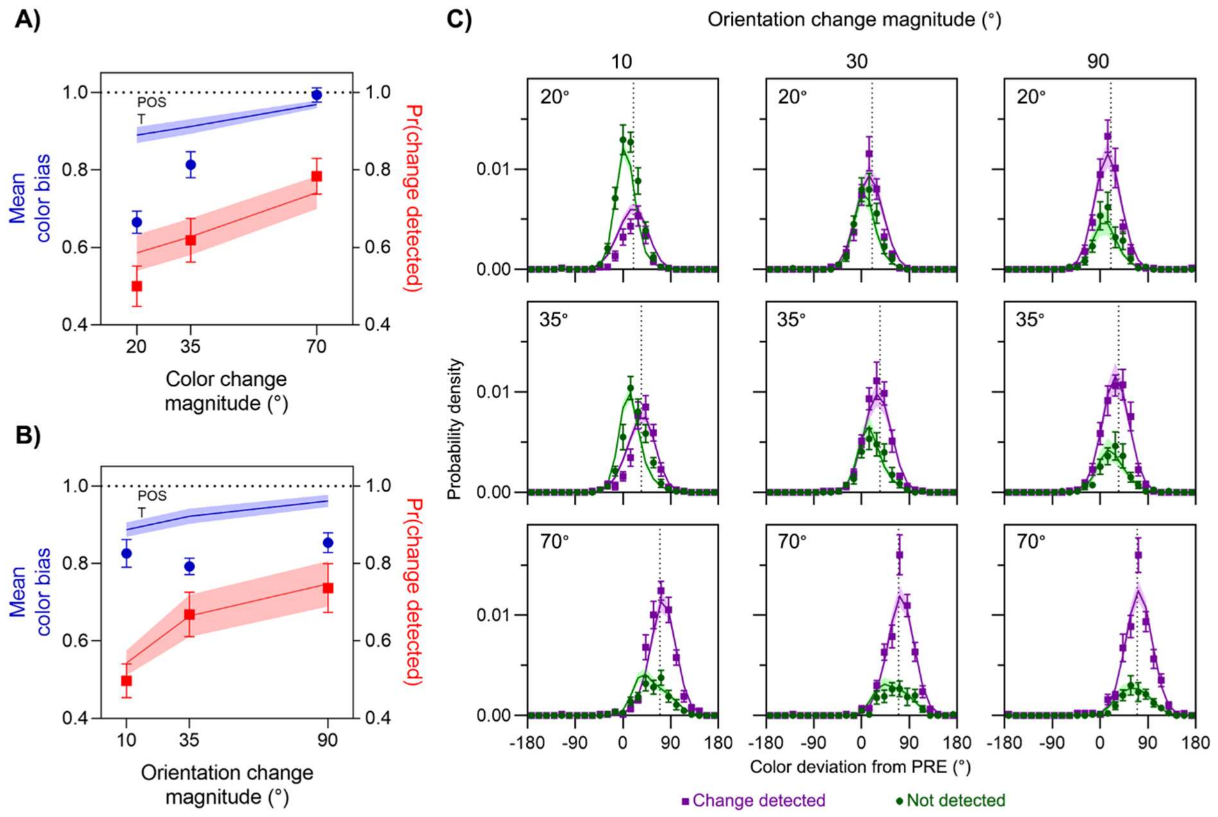

Figure S5. Results from the color-report trials in Experiment 3. (A & B) Mean color bias (blue symbols, left y-axis) and mean frequency of detecting a change (red symbols, right y-axis) are plotted as a function of the magnitude of (A) color change and (B) orientation change. C) Distribution of reported color relative to pre-saccadic (0°) and post-saccadic (dotted line in each panel) values, plotted separately for trials where a change was detected (purple) and not detected (green). Areas under each distribution reflect the frequency of detection. Each panel corresponds to a different pairing of color change (rows: magnitude indicated in top-left of each panel) and orientation change (columns: magnitude indicated at top).

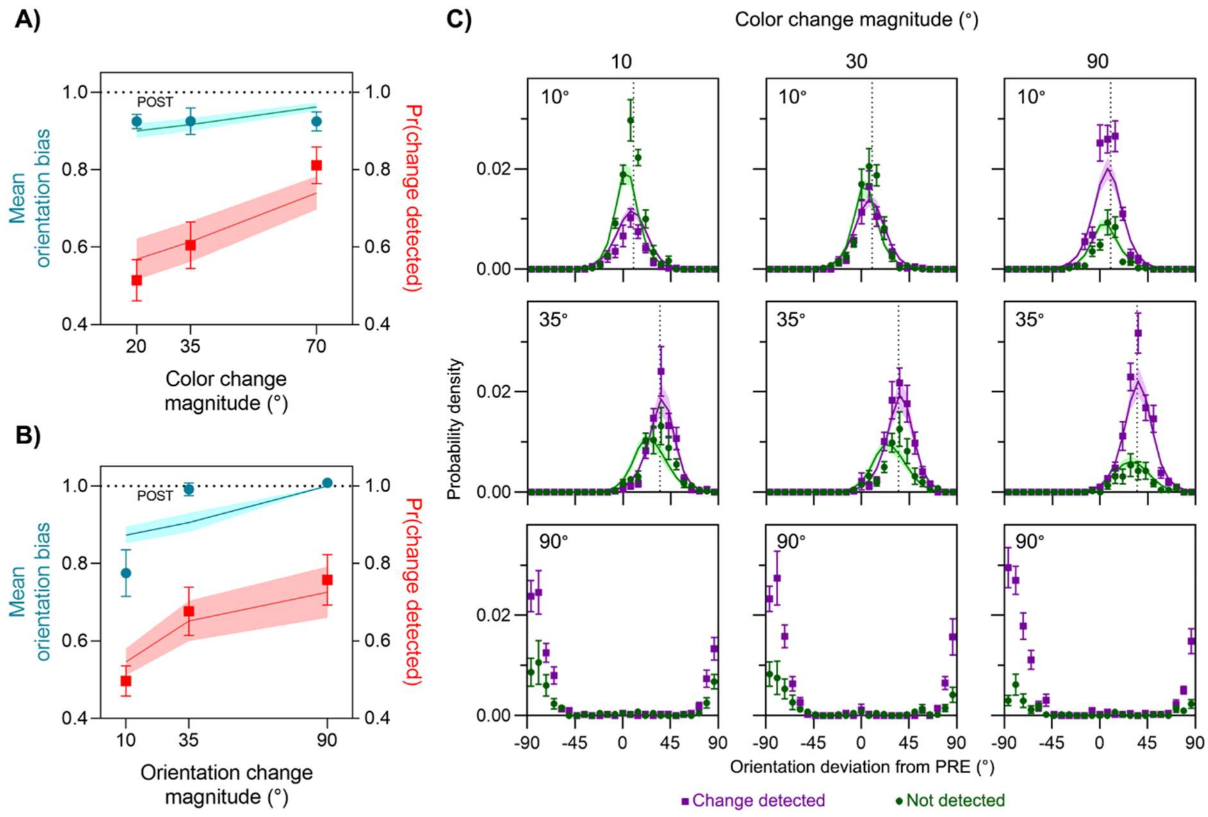

Figure S6. Results from the orientation-report trials in Experiment 3. (A & B) Mean orientation bias (cyan symbols, left y-axis) and mean frequency of detecting a change (red symbols, right y-axis) are plotted as a function of the magnitude of (A) color change and (B) orientation change. (C) Distribution of reported orientation relative to pre-saccadic (0°) and post-saccadic (dotted line in each panel) values, plotted separately for trials where a change was detected (purple) and not detected (green). Areas under each distribution reflect the frequency of detection. Each panel corresponds to a different pairing of orientation change (rows: magnitude indicated in top-left of each panel) and color change (columns: magnitude indicated at top). Model fits for the 90° orientation change condition are not available, as the optimal observer response for such a large change is not uniquely defined.
